# 559. Indocyanine Green Microangiography and Conventional Angiography Demonstrate Similar Tissue Ischemia in Severe Frostbite Injury

**DOI:** 10.1093/jbcr/irag033.299

**Published:** 2026-04-06

**Authors:** Emily Popma, M D MHS, Alexandra M Lacey, Nicholas Larson, Andrew Hartigan, Kristiana Sather, Kirsten A Dalrymple

**Affiliations:** University of Michigan, Ann Arbor, MI; Regions Hospital, Saint Paul, MN; Regions Hospital Burn Center, Minneapolis, MN; Regions Hospital, Saint Paul, MN; University of Minnesota, Saint Paul, MN; Health Partners CCRC, Saint Paul, MN

## Abstract

**Introduction:**

Severe frostbite injury results in thrombosis of microvasculature which can be diagnosed in a variety of ways. The method utilized varies between institutions based on available technologies. There is no current standard imaging technique for the diagnosis of severe frostbite as there have been few direct comparative studies to assess the efficacy of these modalities in a head-to-head manner. Indocyanine green (ICG) microangiography has shown to be a promising imaging technique for the diagnosis of severe frostbite. This study aims to look at a direct comparison between conventional angiography and ICG microangiography in severe frostbite.

**Methods:**

Institutional IRB approval was obtained for this study. Inclusion criteria were: adult patients (≥18 years), diagnosis of severe frostbite injury on conventional angiography, and ability to consent for research participation. Patients underwent serial imaging every 24 hours during the 72 hours of intra-arterial infusions of thrombolytics. If the patient consented to the study, an ICG image was obtained immediately following one of the surveillance angiography images. Angiography images were uploaded to the medical record and ICG images were uploaded to the computer of the PI. A physician from interventional radiology completed a Hennepin Frostbite score noting ischemic tissue on the conventional angiography. Similarly, a physician from the burn service also completed the worksheet noting the ischemic tissue on ICG study.

**Results:**

A total of 8 patients were enrolled in the study over three winters. There was no significant difference in ischemic tissue amount found between the ICG study and the angiography indicating there is correlation between these two studies (*p*=.23) with an average binary agreement of 84.4%. In a Bland–Altman analysis of the two modalities, the scores differed by 0.136 with the ICG study identifying 13.08% more ischemic tissue than the conventional angiography. This study group was small and underpowered for any large-scale conclusions. There were no adverse events noted with the ICG dye being delivered immediately following conventional contrast dye.

**Conclusions:**

This study demonstrates that the conventional angiography and ICG show similar amounts of ischemic tissue with the ICG study possibly detecting more ischemic tissue than angiography. Though this was a small study, this demonstrates that bedside ICG angiography may be equivalent or more sensitive in detecting ischemic tissue in severe frostbite injury.

**Applicability of Research to Practice:**

No single imaging modality has yet been demonstrated to be superior in diagnosing severe frostbite. This study provides more evidence about the use of ICG microangiography in severe frostbite in detecting ischemic tissue.

**Funding for the study:**

N/A.

